# Prolonged duration of surgery is not a risk factor for postoperative complications in patients undergoing total thyroidectomy: a single center experience in 305 patients

**DOI:** 10.1186/s13037-014-0045-2

**Published:** 2014-12-05

**Authors:** Peter C Ambe, Silvia Brömling, Wolfram T Knoefel, Alexander Rehders

**Affiliations:** Department of General and Visceral Surgery (A), University Hospital Düsseldorf, Moorenstr. 5, 40225 Düsseldorf, Germany; Helios Klinikum Wuppertal, Department of Surgery II, Witten – Herdecke University, Heusner Str. 40, 42283 Wuppertal, Germany

**Keywords:** Thyroid surgery, Postoperative hypocalcemia, Recurrent laryngeal nerve injury, Parathormone, Parathyroid glands

## Abstract

**Background:**

Hypocalcemia and nerve injury are the most severe complications after thyroid surgery. The duration of surgery has not been previously considered as a risk factor for postoperative complications in patients undergoing total thyroidectomy. We sort to investigate the influence of prolonged surgery on postoperative complications in patients undergoing total thyroidectomy.

**Methods:**

We hypothesized that a threshold of > 120 minutes of surgical time could represent a surrogate marker for postoperative complications in patients undergoing total thyroidectomy for benign thyroid disorders. The study population was divided into two groups based on the median duration of surgery (120 min): group I ≤ 120 minutes (control group), group II > 120 minutes (study group). The charts of eligible patients undergoing total thyroidectomy within a six-year period from January 1^st^ 2006 to December 31^st^ 2012 were reviewed. The primary outcomes included the rates postoperative hypocalcemia and recurrent laryngeal nerve palsy. The secondary outcomes included the rates of postoperative hemorrhage, wound dehiscence and length of hospital stay.

**Results:**

305 cases of thyroidectomy were included for analysis; 130 (42.6%) control group and 175 (57.4%) study group. Transient (15.4% vs 19.4%) and permanent (3.8% vs. 2.9%) hypocalcemia were recorded in control and study group respectively. The incidence of nerve palsy was 1.5% in the control group and 1.4% in the study group. The mean length of postoperative hospital stay was 3d in both groups. There was no significant difference amongst both groups with regard to postoperative bleeding (p = 0.57) and wound dehiscence (p = 0.31). Prolonged surgery (> 120 min) was not identified as a risk factor for increased postoperative complication.

**Conclusion:**

Prolonged duration of surgery > 120 minutes is not a surrogate marker for postoperative complications in patients undergoing total thyroidectomy.

## Introduction

Hypocalcemia is the most common complication following thyroid surgery. Transient hypocalcemia has been described in over 65% of cases, while permanent post surgical hypocalcemia is thought to occur in up to 10 % of cases [1-9]. Mild postoperative hypocalcemia may be asymptomatic. Severe hypocalcemia however leads to clinical symptoms like Trousseau’s and Chvostek’s signs, paresthesia and muscular cramps [10,11]. Postoperative hypocalcemia does not only increase perioperative morbidity, but may also increase the length of hospital stay, and thus the overall treatment cost. Hemodilution, surgical trauma, removal of one or more parathyroid glands as well as devascularization of the parathyroid glands following ligation of the thyroid vessels have been identified as possible causes of postoperative hypocalcemia [12-15]. Apart from hypocalcemia, recurrent laryngeal nerve injury (RLNI) is the most severe complication following thyroid surgery [16-19]. Besides visual identification of the recurrent laryngeal nerve (RLN), intraoperative monitoring has been shown to reduce the risk of RLNI [20,21].

A review of our thyroid surgery database identified the duration of surgery as a common feature in a number of patients with complications. Prolonged duration of surgery has not previously been examined with regard to postoperative complications in patients undergoing thyroid surgery. The aim of this study therefore was to investigate the effect of the duration of surgery on postoperative complications following total thyroidectomy.

## Patients and methods

Following the approval of the ethic committee of the medical faculty, the charts of patients who underwent thyroid surgery from January 1^st^ 2006 to December 31^st^ 2012 in the Department of General and Visceral Surgery of the University Hospital in Düsseldorf (Germany) were retrospectively reviewed. Only cases of total thyroidectomy were analyzed. Demographic data including age, sex, Body Mass Index (BMI) and comorbidities as defined by the American Society of Anesthesiologists (ASA) were recorded, Table 1. Peri-operative data including the indication for surgery, duration of surgery, postoperative complications, postoperative hospital stay, removal and re-implantation of parathyroid glands were retrieved from surgical documentation sheets, surgeon’s notes and discharge records.

**Table 1:** **Summary of the demographic features of the study population**

**Features**	**Case number**		**P-value**
	**Group (I)**	**Group II**	
Gender (F:M)	112:18	115:60	0.01
Percentage	86.2%:13.8%	65.7%:34.3%	
Age (mean)	55.6 ± 14.3	51.8 ± 14.2	0.28
Range	19 – 84	18 – 79	
BMI < 25.0	55 (42.3%)	68 (38.9%)	0.69
25.1 – 30.0	54 (41.5%)	52 (30.3%)	
30.1 – 35.0	14 (10.8%)	41 (23.4%)	
> 35.0	7 (5.4%)	13 (7.4%)	
ASA 1 – 2	116 (89.2%)	144 (82.3%)	0.09
3 – 4	14 (10.8%)	31 (17.7%)	

Baseline characteristics of the study population.

F: female, M: male, BMI : body mass index (kg/m^2^). Significantly more male patients were included in study group.

We hypothesized that a threshold of > 120 minutes of surgical time could represent a surrogate marker for postoperative complications in patients undergoing total thyroidectomy for benign thyroid disorders. Since our aim was to analyze the effect of the duration of surgery on postoperative complications, potential time consuming procedures like surgery for malignancies, retrosternal, intrathoracic and recurrent goiter were excluded. Furthermore, since both the duration of surgery and surgical outcome are greatly influenced by surgical expertise only procedures led by attending surgeons with expertise in thyroid surgery (more than 200 thyroid procedures) were included for analysis. Because the risk of postoperative hypocalcemia following resection of a single thyroid lobe is low, only cases of total thyroidectomy were included for analysis.

The duration of surgery was defined as the time interval from skin incision to closure in minutes. Since our aim was to investigate the effect of the duration of surgery on postoperative complications, the median duration of surgery for the entire study population (120 min) was chosen as baseline. Based on this time limit, surgery was either classified as normal, i.e. within 120 minutes (group I) or prolonged, i.e. > 120 min (group II).

All patients had a laryngoscopic examination of the vocal cords before and after surgery. The tissue volume was measured preoperatively in all patients via ultrasound. The recurrent laryngeal nerve (RLN) was identified during dissection. Intraoperative neuro-monitoring (IONM) was employed in all cases.

Preoperative and postoperative plasma calcium values were recorded for each patient. As part of our institutional standards, the first postoperative calcium measurement was performed following surgery and then on postoperative day one, if the initial postoperative measurement was not within normal limits. A routine postoperative parathormone measurement was not part of the institutional standards and was only performed if low calcium levels were recorded or following the removal of one or more parathyroid glands.

Hypocalcemia was defined either as blood calcium level below the normal institutional limits (2.10 – 2.43 mmol/l) or following clinical manifestations of hypocalcemia (paraesthesia, muscle spasm, Chvostek’s or Trousseau’s signs) requiring substitution. Recurrent laryngeal nerve dysfunction was diagnosed following vocal cord dysmotility on laryngoscopy in patients with normal laryngoscopic examination prior to surgery. Laryngoscopic examination was performed in all cases on day one or day two following surgery irrespective of the presence or absence hoarseness. As part of our institutional standards, patients were discharge with normal serum calcium levels with or without oral calcium medication, stable wound without clinical and laboratory signs of inflammation.

Data analysis was performed using the Statistical Package for Social Science (SPSS®, IBM Version 21). The results were reported using case numbers and percentages. P – values were calculated using Fischer’s test with levels of significance set at p < 0.05. The results of patients operated upon > 120 min were compared to those operated upon within 120 min (control group) with regard to postoperative hypocalcemia, recurrent laryngeal nerve palsy and length of stay.

## Results

**Figure 1:**
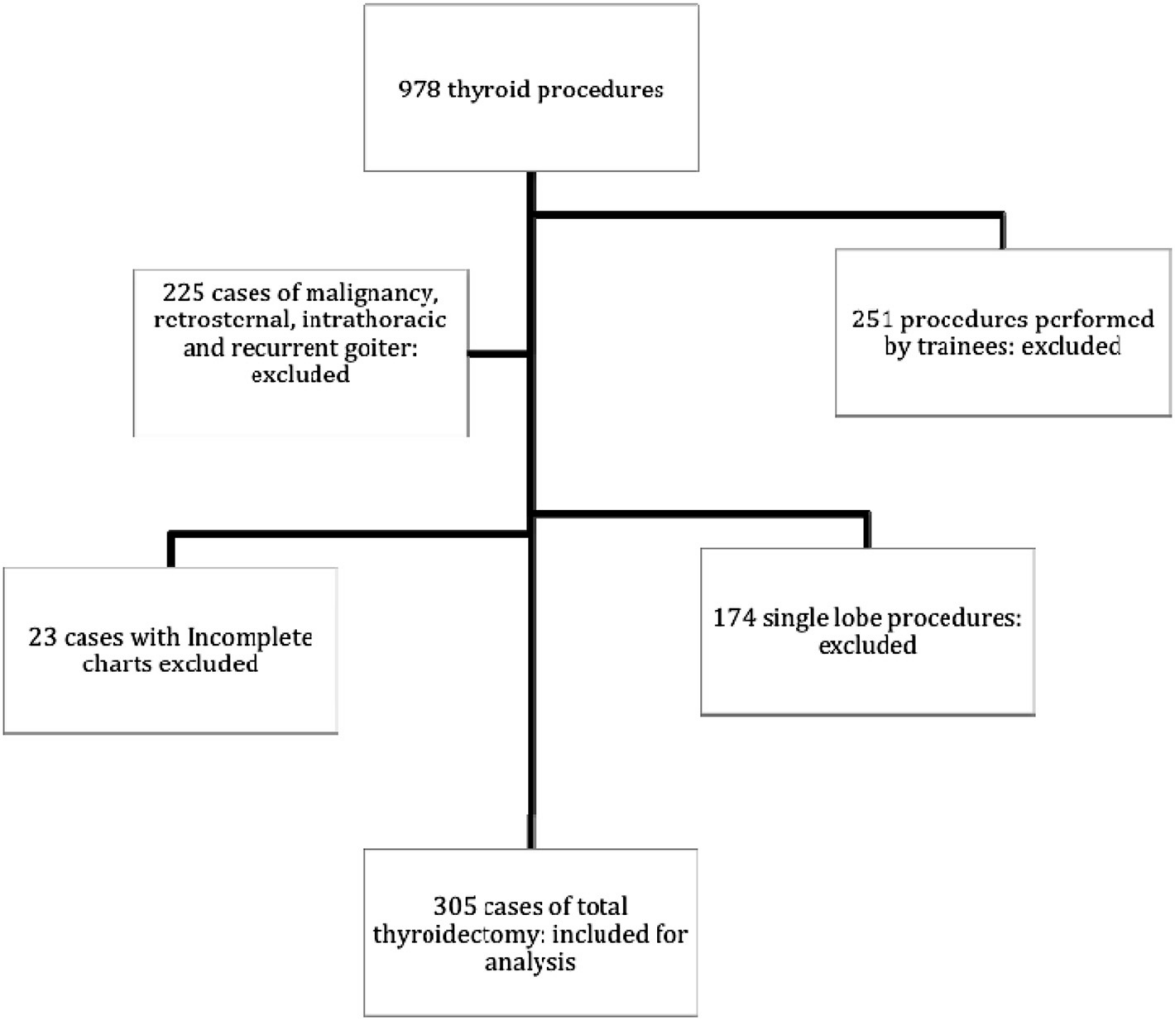
**Distribution of the study population.** 305 cases of total thyroidectomy performed by surgical attendings with experience in thyroid surgery were included for analysis.

The data of 978 patients undergoing thyroid surgery from 2006 to 2012 were reviewed. 305 cases of total thyroidectomy managed by seven surgical attendings with experience in thyroid surgery were included for analysis, Figure 1. The baseline characteristics of the study collective are presented in Table 1, while the perioperative features are presented in Table 2.

**Table 2:** **Summary of clinical features of the study population**

**Features**	**Group I (control)**	**Group II (study)**	**P- value**
Histopathology:			
•multinodular goiter	95 (73.1%)	126 (72.0%)	
•Grave’s Disease	23 (17.7%)	39 (22.3%)	0.91
•Hashimoto’s Disease	12 (9.2%)	10 (5.7%)	
Number of parathyroid glands removed	27/130 (20.8%)	24/175 (13.7%)	0.10
Nerves at risk	260	350	0.56
Mean tissue volume (ml)	38.3 ± 33.5	50.5 ± 41.0	0.03

Perioperative data.

Both groups were comparable with regard to perioperative data.

The mean duration of surgery was 102 ± 14.8 min (range: 55 – 120 min) in group I (control group) and 150 ± 22 min (range: 121 – 204 min) in group II (study group).

The rates of postoperative transient, clinically apparent and permanent hypocalcemia in the general population were 17.7%, 10.8% and 3.2% respectively. Nerve dysfunction was observed in 26 cases. The rates of transient and permanent nerve palsy with regard to the number of lobes resected in this series were 4.3% and 1.5% respectively. Table 3 shows the rates of postoperative complications in both groups.

**Table 3:** **Summary of postoperative complications**

**Characteristics**	**Group I**	**Group II**	**P- value**
Hypocalcemia:			
-transient	20 /130 (15.4%)	34 /175 (19.4%)	0.36
-clinally apparent	13/130 (10.0%)	20/175 (11.4%)	0.69
-permanent	5 /130 (3.8%)	5/175 ( 2.9%)	0.63
Nerve dysfunction			
•transient	8/260* (3.0%)	18 /350* (5.1%)	0.20
•permanent	4 /260* (1.5%)	5/350* (1.4%)	0.32
Postoperative bleeding	4 /130 (3.1%)	3/175 (1.7%)	0.57
Wound dehiscence	1 /130 (0.8%)	4/175 (2.3%)	0.31

Postoperative complications.

*Represents the number of thyroid lobes resected. There was no significant difference amongst both groups with respect to postoperative complications.

Parathyroid gland removal was recorded in 51 cases (51/305 = 16.7%). Re-implantation of parathyroid glands was recorded in 11 cases in group I and 13 cases in group II. There was no significant difference in the number of parathyroid glands removed and re-implanted in both groups. Equally, there was no significant difference between both groups with respect to parathyroid hormone levels.

The overall rate of morbidity in this series was 30.2%. There was no mortality in this series.

The mean length of postoperative hospital stay in the entire collective was three days. This corresponded to 3.5 d in group I and three days in group II respectively.

## Discussion

Thyroid surgery is one of the most common endocrine surgical procedures. Postoperative hypocalcemia and recurrent laryngeal nerve palsy represent the most severe complications following thyroid surgery. Recurrent, retrosternal and intrathoracic goiter as well as malignancies of the thyroid gland have been identified as possible risk factors for postoperative complications [22-25]. Prolonged duration of surgery however has not been considered as a risk factor for postoperative complications.

The aim of this study was to investigate whether or not the duration of surgery could be a surrogate marker for postoperative complications in patients undergoing total thyroidectomy. Based on the median duration of surgery of 120 minutes for total thyroidectomy in our department, the study population was divided into two groups based on the length of surgery. Group I comprised of cases with surgery lasting ≤ 120 min while group II included cases with surgery > 120 min. Procedures lasting > 120 minutes were considered “prolonged”. Only cases managed by surgical attendings with expertise in thyroid surgery were included in this series.

Three hundred and five cases of total thyroidectomy were included for analysis. Based on the above mentioned definition of the duration of surgery, 175 cases of “prolonged” total thyroidectomy were identified and compared to 130 cases of total thyroidectomy performed within 120 minutes. With the exception that significantly more male patients were managed in the study group, both groups were comparable in terms of baseline characteristics, diagnosis and comorbidities.

The rates of transient, clinically apparent and permanent postoperative hypocalcemia in this series were comparable with existing data [3,6,26-28]. Prolonged surgery was not associated with an increased risk of postoperative hypocalcemia. Equally, the risk of injury to the recurrent laryngeal nerve was not significantly higher in patients with prolonged surgery. Furthermore, there was no difference in the length of postoperative hospital stay amongst both groups.

The mean thyroid volume measured using ultrasound sonography prior to surgery was significantly higher in the study group compared to the control group (50 vs. 38 ml). This may in part explain the difference in the duration of surgery, considering the fact that large goiters could be more challenging to manage. Furthermore, significantly more male patients were included in the study group compared to the control group. One may argue that male patients might present a more difficult situs, thus the longer duration of surgery. However, the current literature does not suggest the male gender to be a risk factor for the development of hypocalcemia following thyroidectomy [29].

Besides the larger volume of the thyroid gland, it is difficult to explain why surgery lasted longer in the study group. Both groups were comparable in terms of baseline characteristics, diagnosis and ASA scores. All procedures were performed by experienced surgeons, no single surgeon was identified as generally “slow” and the operative records failed to point out any form intraoperative dissection difficulties.

We could not identify patient specific factors, which could have led to an increase in the duration of surgery in the study group. However, some operation room and surgeon specific properties warrant discussion. First being a teaching hospital, all attending surgeons are actively involved in surgical training. Surgical teaching requires patience from both the teacher and the trainee and may be time consuming. Besides, the attending’s attention or opinion might be needed by a junior surgeon (resident or fellow) in the next operation room. Second, it is rather a normal occurrence, that the senior surgeons may interrupt dissection to answer calls on important issues concerning patients (in the wards), administrative or research issues. These may to some degree have influenced the duration of surgery.

Taken together, our results suggest that the duration of surgery is not a surrogate marker for postoperative hypocalcemia in patients undergoing total thyroidectomy. Equally, the risk of recurrent laryngeal nerve palsy was not increased in patients with prolonged duration of surgery. Besides departmental issues, no patient specific factors could be identified as the cause of prolonged surgery in the study group.

### Limitations

This study is limited by its retrospective nature. Important information about surgical difficulty associated with dissection, which in part could have explained the longer duration of surgery was not mentioned in the operative records. The difference in the speed of surgery could not be justified. Furthermore, the results reported in this series represent the findings from a single center and cannot be generally projected on other centers. Therefore, the trends reported in this series must be interpreted with caution.

## Conclusion

Prolonged duration of surgery > 120 minutes is not a surrogate marker for postoperative complications in patients undergoing total thyroidectomy.
